# *C-terminally encoded peptide-*like genes are associated with the development of primary root at *qRL16.1* in soybean

**DOI:** 10.3389/fpls.2024.1387954

**Published:** 2024-04-15

**Authors:** Giriraj Kumawat, Dong Cao, Cheolwoo Park, Donghe Xu

**Affiliations:** ^1^ Japan International Research Center for Agricultural Sciences, Tsukuba, Ibaraki, Japan; ^2^ Crop Improvement Section, ICAR-Indian Institute of Soybean Research, Indore, Madhya Pradesh, India; ^3^ Key Laboratory of Biology and Genetic Improvement of Oil Crops, Ministry of Agriculture and Rural Affairs, Oil Crops Research Institute, Chinese Academy of Agricultural Sciences, Wuhan, Hubei, China

**Keywords:** primary root, C-terminally encoded peptides, root length, genes, soybean

## Abstract

Root architecture traits are belowground traits that harness moisture and nutrients from the soil and are equally important to above-ground traits in crop improvement. In soybean, the root length locus *qRL16.1* was previously mapped on chromosome 16. The *qRL16.1* has been characterized by transcriptome analysis of roots in near-isogenic lines (NILs), gene expression analysis in a pair of lines contrasting with alleles of *qRL16.1*, and differential gene expression analysis in germplasm accessions contrasting with root length. Two candidate genes, *Glyma.16g108500* and *Glyma.16g108700*, have shown relatively higher expression in longer root accessions than in shorter rooting accessions. The C-terminal domain of *Glyma.16g108500* and *Glyma.16g108700* is similar to the conserved domain of C-terminally encoded peptides (CEPs) that regulate root length and nutrient response in Arabidopsis. Two polymorphisms upstream of *Glyma.16g108500* showed a significant association with primary root length and total root length traits in a germplasm set. Synthetic peptide assay with predicted CEP variants of *Glyma.16g108500* and *Glyma.16g108700* demonstrated their positive effect on primary root length. The two genes are root-specific in the early stage of soybean growth and showed differential expression only in the primary root. These genes will be useful for improving soybean to develop a deep and robust root system to withstand low moisture and nutrient regimes.

## 1 Introduction

Soybean (*Glycine max* L. Merill) is an important crop grown over an area of 133.8 Mha with a total production of 348.86 Mt during 2022 (https://www.fao.org/faostat/). Soybeans are used in oil, food, feed, nutraceuticals, biofuel, and several non-food industries. Soybean originated in China, and over the period it spread to the American and Asian continents (Hymowitz, 1970). The developmental biology and genetics of the above-ground parts of soybean have been well characterized, and information on the below-ground parts is slowly emerging. Roots are essential organs of plants for anchoring and fulfilling nutritional requirements, and show remarkable plasticity in response to environmental cues. Root architecture plays an important role in the robust performance of the whole plant by the efficient acquisition of water and nutrients from the soil. To decipher the genetic architecture of root architecture traits in soybean, several quantitative trait loci (QTLs) have been mapped for various root traits (Abdel-Haleem et al., 2011; Brensha et al., 2012; Liang et al., 2014; Manavalan et al., 2015; Prince et al., 2015; Prince et al., 2019; Yang et al., 2019; Prince et al., 2020; Chen et al., 2021; Wang et al., 2022). However, only a few genes have been characterized to control the characteristics of root architecture in soybean. Soybean *Xyloglucan endoglycosylase/hydrolase 1* (*GmXTH1*) overexpression showed higher root phenotypic traits, including main root length, lateral root length, root volume, root dry weight, and root fresh weight in transgenic soybean plants compared to wild-type plants (Song et al., 2022). Similarly, overexpression of two soybean transcription factors, *GmNAC19* and *GmGRAB1*, significantly increased root growth and biomass in transgenic composite plants (Mazarei et al., 2023). Interestingly, the overexpression of *GmNAC19* also enhanced water stress tolerance in transgenic composite plants.

In *Arabidopsis*, genes such as *ROOT GROWTH FACTOR*, *SHORT-ROOT, SCARECROW, AUXIN1 (AUX1) family*, or efflux carriers of the *PIN-FORMED (PIN) family, EXOCYST70A3, COMPACT ROOT ARCHITECTURE*, and *C-TERMINALLY ENCODED PEPTIDES (CEPs)*, are important genes that regulate root development and nutrient uptake response (Benfey et al., 1993; Scheres et al., 1995; Luschnig et al., 1998; Ruzicka et al., 2007; Swarup et al., 2007; Ohyama et al., 2008; Matsuzaki et al., 2010; Delay et al., 2013; Huault et al., 2014; Ogura et al., 2019). In our previous study, a major locus, *qRL16.1*, was identified to control the length of primary root in soybean (Chen et al., 2021). In our current study, we found that the candidate gene for locus *qRL16.1* have similarity to *Arabidopsis thaliana* C-terminally encoded peptides (CEPs). CEPs are a class of small post-translationally modified signaling peptides that are involved in the regulation of root development (Ohyama et al., 2008; Delay et al., 2013; Roberts et al., 2013). The mature peptide encoded by the *CEP* genes is a 15 amino acid peptide sequence containing two post-translationally modified hydroxyproline residues and is found in the conserved C-terminal domain (Ohyama et al., 2008). CEPs also act as a root-to-shoot mobile peptide hormone that triggers systemic nitrogen acquisition under nitrogen-starved roots in *Arabidopsis* (Ohyama et al., 2008; Tabata et al., 2014; Ohkubo et al., 2017).

The purpose of this study was to characterize the transcriptome of *qRL16.1* and identify best candidate genes through differential expression analysis in soybean germplasm contrasting for primary root length, and association analysis. Synthetic peptide assay and tissue specific gene expression analysis were employed to further support functionality and root specificity of the identified candidate genes. This research will help in improving the soybean to develop a deep root system that can withstand low moisture and nutrient regimes.

## 2 Materials and methods

### 2.1 Plant material

K099 (shorter primary root) and Fendou 16 (longer primary root) are parental genotypes of the mapping population used to identify the *qRL16.1* a primary root length locus (Chen et al., 2021). Residual heterozygous line-derived near-isogenic lines (NILs): Near-isogenic lines differing at *qRL16.1*, which were selected from the same recombinant inbred line (RIL) but heterozygous at *qRL16.1* (Chen et al., 2021). NIL-F had the Fendou 16 homozygous genotype, and NIL-K had the K099 homozygous genotype at *qRL16.1*. Advanced backcross lines BC4-F and BC4-K: BC4-F had the Fendou 16 homozygous genotype and BC4-K had the K099 homozygous genotype at the mapped *qRL16.1* region in the background of K099 (Chen et al., 2021). A total of 76 germplasm accessions from a mini-core collection of the world soybean germplasm (Kaga et al., 2012), together with K099 and Fendou16, were used for phenotyping and analysis of the association of root traits with identified polymorphisms.

### 2.2 RNA-seq of NILs

Root samples of NIL-K and NIL-F were collected from 7-day-old seedlings. Each sample comprised roots collected from five individual plants, and three biological replicates were analyzed. Total RNA was extracted from each sample and cDNA was synthesized with adapters and sequenced using the Illumina HiSeq 2500 analyzer at BGI Technologies (Shenzhen, China). The adapter reads and low-quality reads (bases with quality values ≤ 5) were filtered out of the raw data to obtain high-quality (clean) reads, which were mapped to the soybean reference genome (*G. max* Wm82.a2.v2) downloaded from Phytozome (Goodstein et al., 2012) using HISAT v2.0.4 (http://www.ccb.jhu.edu/software/hisat). Significant differentially expressed genes (DEGs) were obtained with fold changes ≥ 2.0 or ≤ −2.0 for up- or down-regulated genes and adjusted *P*-value ≤ 0.05.

### 2.3 Sequencing and real-time PCR analysis of the DEGs

Genomic sequences containing coding sequences and upstream promoter regions of two DEGs, *Glyma.16g108500* and *Glyma.16g112400*, were sequenced from both parents, i.e., K099 and Fendou16, using Sanger sequencing. For real-time PCR based expression analysis of DEGs, primary root tip and lateral root tissues from K099, Fendou16, NIL-K, NIL-F, BC4-F, and BC4-K were collected from 3-week-old plants and frozen in liquid nitrogen. RNA isolation from three biological replicates of each sample was performed using the RNeasy plant mini kit (Qiagen, Germany). The isolated RNA was treated with DNAse I using the RapidOut DNA Removal Kit (Thermo Scientific, USA). Purified RNA was used for cDNA synthesis using the PrimeScript RT master mix (TAKARA BIO, Japan). Comparative quantitative real-time PCR (RT-PCR) was performed using Powerup SYBR Green Master mix (Applied Biosystems, USA) for selected candidate genes. *GmActin* (*Glyma.18g290800*) was used as an internal reference gene. The sequences of primers used for RT-PCR are given in **Table 1**. The 2^-ΔΔCT^ method was used for analyses of relative gene expression levels (Livak & Schmittgen (2001).

**Table 1:** Primers used for gene expression studies using real-time PCR.

Primer name	Sequence (5’ > 3’)
GmActin-F	ATCTTGACTGAGCGTGGTTATTCC
GmActin-R	GCTGGTCCTGGCTGTCTCC
*Glyma.16g108500*-F	ACCTTGTGTTGTGCTCTGGT
*Glyma.16g108500*-R	AGCATGTGGTGGGTGAATGT
*Glyma.16g112400*-F	ACCACTACACATTGGGTTTGGAT
*Glyma.16g112400*-R	CTTCCTCACTCTCCCCTGGAT
*Glyma.16g108400*-F	GGTGATGCTTACCGCCCAA
*Glyma.16g108400*-R	TGGTGTTTCATGCCCCACA
*Glyma.16g108700*-F	TGGAAGCATCAAGAAAGCTCA
*Glyma.16g108700*-R	GGTGTACCAAAGTGCAGGAG

### 2.4 Phenotyping of world core collection of soybean germplasm

A total of 76 accessions from the world soybean mini-core collection of Japan, along with K099 and Fendou16, were used for phenotyping of primary root length, total root length, root volume, surface area, and root tips using hydroponic culture (Chen et al., 2021). The roots of 15-day-old plants (*n* = 4) were scanned and analyzed using WinRHIZO software (Regent Instruments Inc., Canada). Phenotyping data from these accessions were used for the marker-trait association analysis. The top four accessions for longer primary roots and the top four accessions for shortest primary roots were used for the validation of four differentially expressed genes.

### 2.5 Genotyping of SNP and Indel in the germplasm

Accessions of the world soybean mini-core collection of Japan, together with K099 and Fendou16, were genotyped with one SNP (RL1SNP968_T/C) and one Indel (RL1Indel363_–/AA) marker selected from the promoter region of *Glyma.16g108500*. Allele-specific PCR primers were used for the amplification of specific SNP or Indel alleles and were separated on 8.0% polyacrylamide gel. The DNA band pattern was visualized using a Pharos FX™ Molecular Imager (Bio-Rad, Tokyo, Japan) instrument for scoring alleles. The Welch’s t-test was used to test the (null) hypothesis that two populations formed by alternate alleles have equal means.

### 2.6 *CEP-*like genes in soybean

In order to identify *CEP-*like genes in soybean, protein sequences from 16 known *CEP* genes of *Arabidopsis thaliana* were used as queries in a BLASTP search against the Williams 82 genome Wm82.v2 in SoyBase (www.soybase.org). The 14 homologs identified in the soybean genome were used again for iterative BLASTP to identify more *CEP-*like genes in the soybean genome. Thus, 23 more matching gene sequences were identified. The 37 matching sequences were manually filtered for proteins with an N-terminal signal peptide sequence for the secretory pathway and a conserved C-terminal ‘CEP-like’ sequence motif ‘FRPToPGHSPGVGH’ (at 70% consensus cutoff), using multiple sequence alignment by CLUSTALW and motif prediction by MEME Suite tool (Bailey et al., 2015). Finally, 25 *CEP-*like genes were selected and used for phylogeny and motif analysis. MEGA11 software was used to construct the phylogeny of 25 *CEP-*like gene protein sequences (Tamura et al., 2021). The neighbor-joining clustering method with 10,000 bootstrap iterations was used to build a consensus phylogenetic tree. The MEME Suite tool was used for consensus motif detection with the following settings: motif site distribution as any number of sites per sequence, motif width 6 to 50, minimum site for motif 2, and maximum number of motifs of 10 (Bailey et al., 2015).

### 2.7 Synthetic peptide assay

The conserved regions of CEP for *Glyma.16g108500* and its homolog *Glyma.16g108700* were predicted, and putative post-translational modifications of proline amino acid residues, that is, hydroxyproline, were included for synthesis (**Table 2**). Synthetic peptides *Glyma.16g108500* (CEP-RL1) and *Glyma.16g108700* (CEP-RL2) were synthesized artificially at Eurofins, Japan. The seeds of the short-rooting genotype K099 were sown in vermiculite and young seedlings 4 days after sowing were used for the synthetic peptide assay. The 4-day seedlings were transferred to 0.5× Hoagland solution (pH 6.5). For each treatment, eight seedlings were placed in a plastic box (13 cm × 8.5 cm × 6 cm) containing 500 ml Hoagland solution. The outer surface of the box was covered with aluminum foil to prevent root exposure to the light. Following concentrations were used for the treatment in Hoagland solution: i) Control: No CEP; ii) CEP-RL1: 1 µM; iii) CEP-RL1: 2 µM; iv) CEP-RL2: 1 µM; v) CEP-RL2: 2 µM. The seedlings were incubated in an incubation chamber kept at 22° C night and 24°C day temperatures and supplied with 14 h of fluorescent light. Roots were harvested after 10 days of peptide treatment and scanned for recording of root traits using WinRHIZO (Regent Instruments Inc., Canada). The recorded root traits were primary root length, total root length, and number of root tips.

**Table 2:** Sequences of synthetic peptides of *Glyma.16g108500* and *Glyma.16g108700* CEPs.

Gene	Peptide name	Peptide sequence
*Glyma.16g108500*	CEP-RL1	DAFR{HYP}TS{HYP}GHS{HYP}GVGH
*Glyma.16g108700*	CEP-RL2	DAFR{HYP}TCRGHS{HYP}GAGH

### 2.8 Tissue-specific gene expression of *Glyma.16g108500* and *Glyma.16g108700*

Two time stages, 4-day, and 7-day, were selected for gene expression analysis of *Glyma.16g108500* and *Glyma.16g108700* in parental genotypes K099 and Fendou16. Tissues were collected at 4 days after sowing, from the shoot apex (2 mm) and primary root tip (2 cm), and at 7 days after sowing, from shoot apex (2 mm), the primary root tip (2 cm), and lateral root tips (2 cm), for tissue-specific gene expression analysis.

## 3 Results

### 3.1 Identification of DEGs for *qRL16.1* by root transcriptome analysis

RNA-Seq analysis of the root transcriptome of two NILs identified total 317 genes upregulated and 192 downregulated in NIL-F compared to NIL-K (**Supplementary Figure 1A**). Differential expression analysis for genes from the genomic region of primary root length QTL *qRL16.1* (Chr.16:27158682.28953489), among two NILs, identified only two genes, *Glyma.16g108500* and *Glyma.16g112400*, showing a significant fold difference in their expression levels (**Supplementary Table 1**). The gene expression analysis by RT-PCR identified that the expression of *Glyma.16g108500* was 3.0 fold higher in Fendou16 tissues compared to K099 tissues, and it was 4.47 fold higher in NIL-F tissues than in NIL-K tissues. Conversely, the expression level of *Glyma.16g112400* was 18.11 fold lower in Fendou16 tissues compared to K099 tissues, and it was 7.21 fold lower in NIL-F tissues than in NIL-K tissues (**Supplementary Figures 1B, C**). The genomic sequence of two DEGs, *Glyma.16g108500* and *Glyma.16g112400*, including a 2-kb promoter region, was sequenced from both parents, Fendou16 and K099. No sequence differences were identified in the coding DNA sequence of both genes between the two parents. Upstream 2-kb sequencing of *Glyma.16g108500* identified two base insertions at the 363 position (–/TA), a single nucleotide polymorphism (SNP) at -968 (T/C), and a single base insertion at the 1038 position (A) in Fendou 16. The 2 kb upstream sequencing of *Glyma.16g112400* did not detect any sequence difference between the two parental genotypes.

### 3.2 Gene annotations and homology search identified *Glyma.16g108500* as a *CEP-*like gene in soybean

According to gene annotations assigned in SoyBase (www.soybase.org), *Glyma.16g108500* was annotated as an uncharacterized protein, root development, and DNA-directed RNA polymerase II subunit RPB1-like protein, while *Glyma.16g112400* was annotated as an uncharacterized protein. Furthermore, the analysis of protein homology in the InterPro protein database identified that *Glyma.16g108500* encodes a C-terminally encoded peptide (CEP), a C-terminally conserved secreted protein in *Arabidopsis* (https://www.ebi.ac.uk/interpro/entry/InterPro/IPR033250/). Based on protein homology, two more paralogs of *Glyma.16g108500* were also identified in close proximity to *Glyma.16g108500* in the genomic region of *qRL16.1*. These two paralogs, *Glyma.16g108700* and *Glyma.16g108400*, showed 72% and 80% homology of the protein sequence with *Glyma.16g108500*, respectively.

### 3.3 Validation of identified DEGs in parents, NILs, and advanced backcross lines

**Figure 1:**
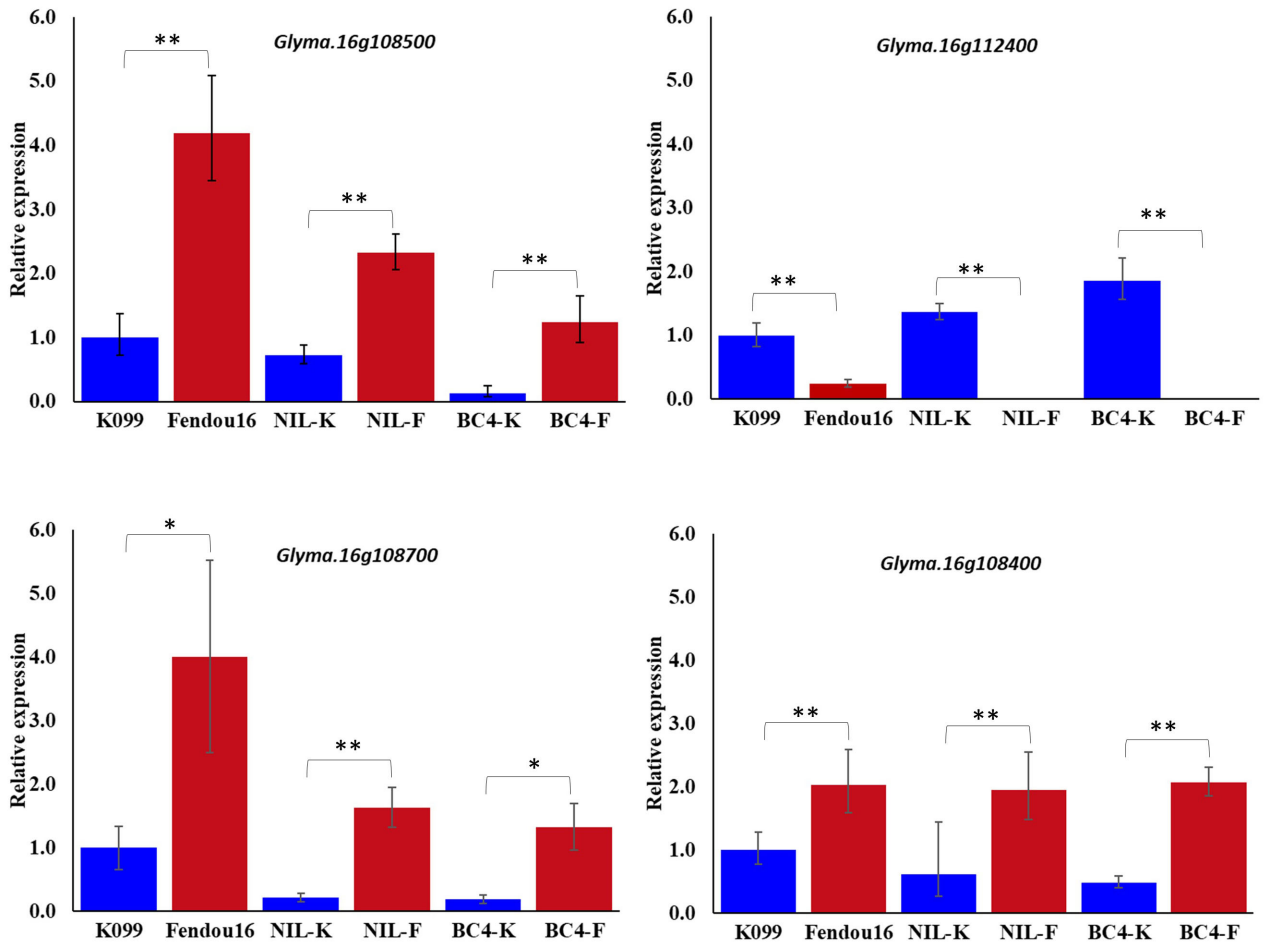
Relative expression of *Glyma.16g108500*, *Glyma.16g112400, Glyma.16g108700* and *Glyma.16g108400*, at 3 weeks primary root tips of K099, Fendou 16, NILs and BC_4_ backcross lines. *GmActin* was used as a normalizer. Significant differences between K099 and Fendou 16, NIL-K and NIL-F, BC4-K and BC4-F, are marked with asterisks (*P<0.05; **P<0.01; Student’s t-test).

The two genes *Glyma.16g108500* and *Glyma.16g112400* were validated for differential expression by real-time PCR in primary root tip of two parents, a pair of contrasting NILs, and a pair of contrasting BC_4_ backcross lines. Expression analysis identified that *Glyma.16g108500* showed higher expression in longer rooting genotypes, whereas *Glyma.16g112400* showed higher expression in shorter rooting genotypes (**Figure 1**). In the case of *Glyma.16g108500*, although the expression fold difference in the contrasting alleles NILs and BC_4_s was similar to that of the parents, the level of expression in both pairs of contrasting lines was slightly reduced compared with that in the two parents. The analysis of gene expression for two *Glyma.16g108500* paralogs, *Glyma.16g108700* and *Glyma.16g108400*, among three pairs of contrasting genotypes identified that *Glyma.16g108700* and *Glyma.16g108400* also showed higher expression in longer rooting genotypes; however, the level of expression fold difference was relatively higher in the case of *Glyma.16g108700* (**Figure 1**).

### 3.4 Phenotyping of the world soybean mini-core collection for root traits

The descriptive statistics of different root traits in 76 soybean accessions, Fendou 16 and K099, at 15 days of growth are given in **Table 3**. The primary root length ranged from 16.3 cm in CHUUHOKU 2 to 51.45 cm in BARITOU 3A, with a mean root length of 34.4 cm. The total root length varied from 142.8 cm to 606.4 cm with a mean value of 402.7 cm.

**Table 3:** Descriptive statistics of root traits in 78 soybean accessions of the soybean mini core collection (Japan).

	Primary root length (cm)	Total root length (cm)	Total root volume (cm^3^)	Total root tips	Root surface area (cm^2^)
Minimum	16.30	142.87	0.41	60.75	27.15
Maximum	51.45	606.48	1.74	450.50	108.53
Mean	34.40	402.76	0.98	257.46	69.86
Standard deviation	6.41	111.87	0.29	82.79	18.85
Variance	41.09	12515.11	0.09	6854.27	355.19

**Figure 2:**
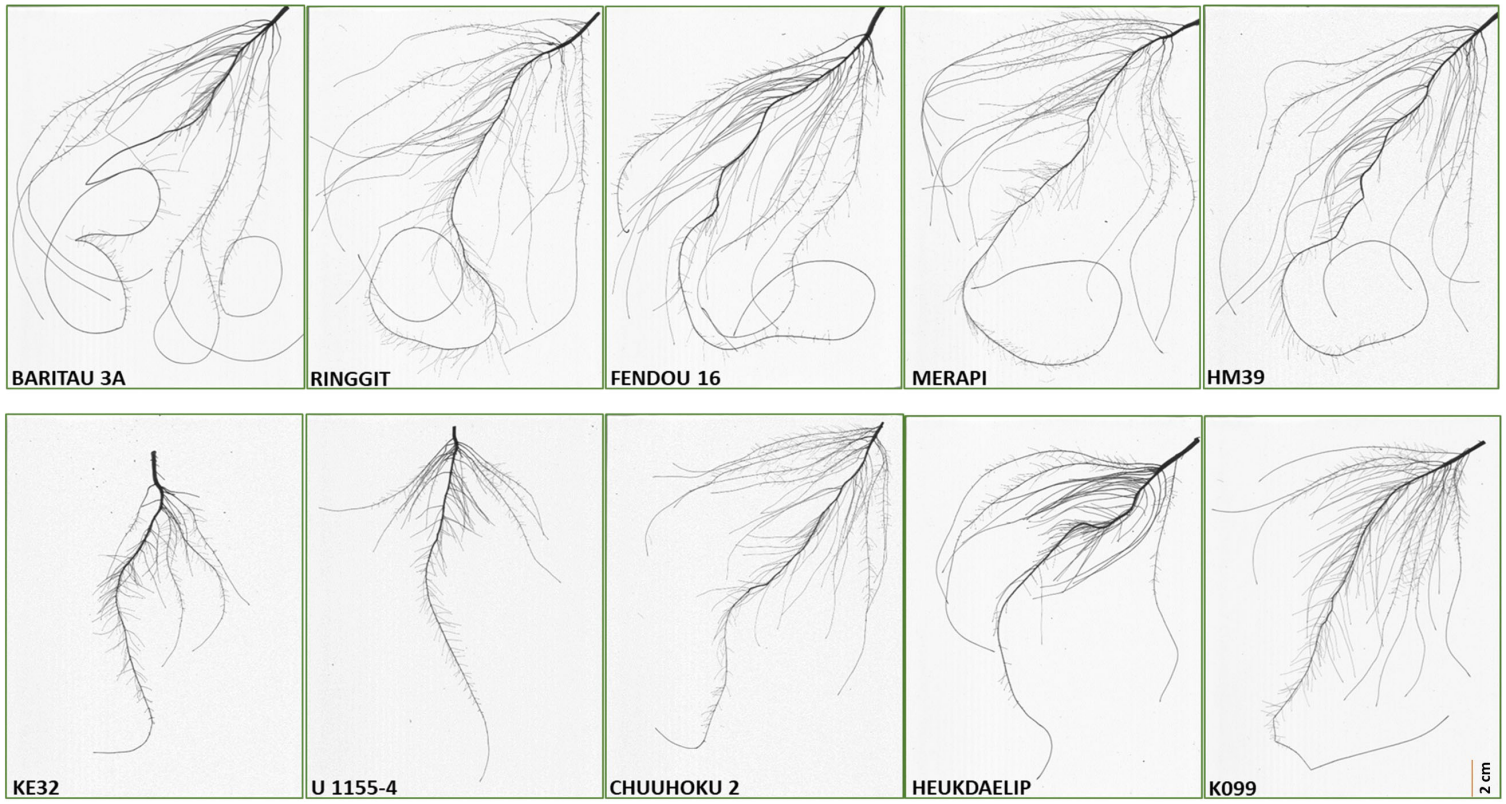
Root scan pictures of the top four contrasting root length genotypes along with K099 and Fendou 16.

The five high and five low contrasting accessions for primary root length and total root length traits along with Fendou 16 and K099 were again evaluated. In this confirmation experiment, similar results were obtained for short- and long-rooting genotypes. Among the top five accessions for primary root length, BARITOU 3A showed the highest primary root length of 53.1 cm, followed by RINGGIT (51 cm) and Fendou 16 (49.2 cm). From this experiment, the top four accessions for longer primary root and the four best accessions for short primary root were selected for validation of four differentially expressed genes (**Table 4**, **Figure 2**).

**Table 4:** Primary root length and total root length values of top four contrasting accessions along with K099 and Fendou 16.

	Genotypes	Primary root length (cm)	Total root length (cm)
Top five longest rooting type	BARITOU 3A	53.12	475.48
	RINGGIT	51.05	552.40
	Fendou 16	49.25	468.78
	MERAPI	47.78	636.60
	HM39	46.20	489.32
Top four shortest rooting genotypes along with K099	KE32	23.43	229.18
	U 1155-4	25.38	422.28
	CHUUHOKU 2	25.50	367.38
	HEUKDAELIP	28.85	390.67
	K099	37.37	523.68

### 3.5 Validation of identified DEGs in contrasting root-length germplasm

**Figure 3:**
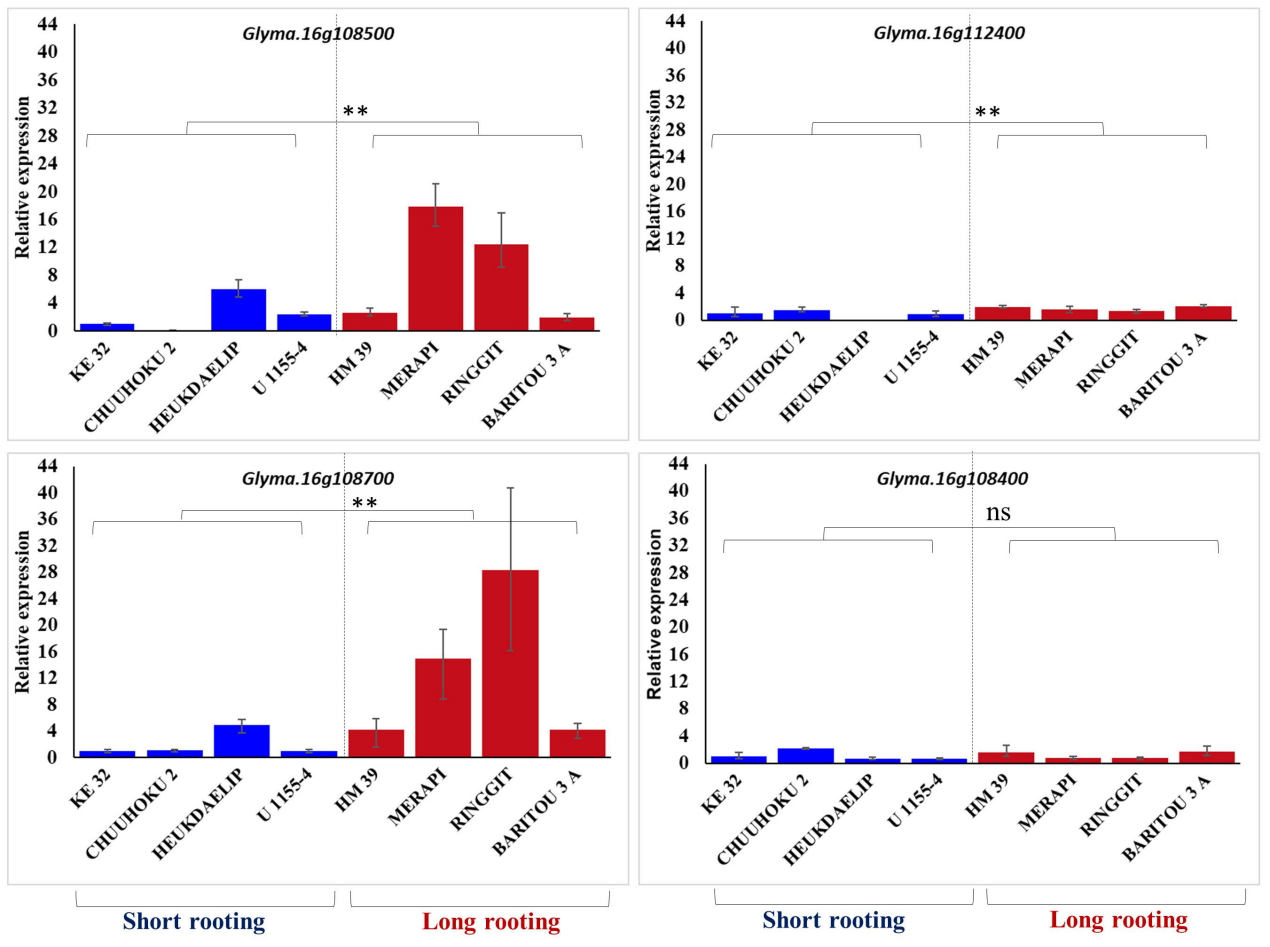
Relative expression of *Glyma.16g108500*, *Glyma.16g112400*, *Glyma.16g108700*, and *Glyma.16g108400* in the four genotypes with the longest and shortest rooting genotypes. *GmActin* was used as a normalizer. Significant differences between short rooting and long rooting genotype sets are marked with asterisks (**P<0.01; ns, not significant; Student’s t-test).

The four differentially expressed genes identified among three contrasting pairs of high and low root length lines from the genomic region of *qRL16.1* were further analyzed for their expression in the top four long rooting and top-four shortest rooting germplasm selected from the world core collection of soybean. Gene expression analysis using real-time PCR showed that *Glyma.16g108500* and *Glyma.16g108700* had relatively higher expression in longer rooting genotypes than in shorter rooting accessions, except for accession Heukdaelip (**Figure 3**). However, *Glyma.16g108400* and *Glyma.16g112400* did not show differential expression between the two contrasting groups in accordance with root length and expression pattern observed in the NILs, thus demeaning their role in the regulation of the development of primary root in soybean (**Figure 3**).

### 3.6 Association analysis in world soybean mini-core collection germplasm

**Figure 4:**
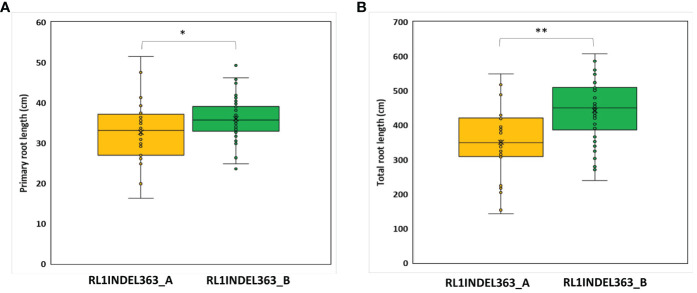
Genotypic association for RL1INDEL363_–/TA alleles with root traits in 78 accessions of the mini core collection of Japan **(A)** Primary root length, **(B)** Total root length. RL1INDEL363_A represents the K099 allele and RL1INDEL363_B represents the Fendou 16 allele. * and ** show significant at *P* < 0.05 and *P* < 0.001 in the Welch’s t-test, respectively.

Coding region of both DEGs, *Glyma.16g108500* and *Glyma.16g108700*, was sequenced but no polymorphism was detected between K099 and Fendou 16. The two polymorphisms, SNP at -968 (RL1SNP968_T/C) and Indel at -363 (RL1INDEL363_–/TA), identified in the upstream sequence of *Glyma.16g108500*, were analyzed in the germplasm to test the association of allelic variation present in the proximity of two DEGs identified in contrasting germplasm. Genotyping of the RL1SNP968_T/C and RL1INDEL363_–/TA markers showed identical marker genotypes among 78 genotypes with 36% and 64% allele frequencies for the K099 and Fendou16 alleles, respectively. The Welch *t*-test showed significant differences in primary root length and total root length between two populations formed by alternate alleles of RL1INDEL363_–/TA (**Table 5**, **Figure 4**).

**Table 5:** Welch’s t-test for RL1INDEL363_–/TA allele association with root length traits in 78 germplasm accessions.

RL1INDEL363_--/TA allele	Primary root length (cm)		Total root length (cm)	
	Allele A	Allele B	Allele A	Allele B
Mean	32.62	36.02	349.88	442.33
Variance	60.99	26.47	12218	8225
Observations (*N*)	28	50	28	50
t-Statistics	-2.066		-3.771	
*P*(T<=t) one-tail	0.02267		0.00022	
t Critical one-tail	1.6838		1.6779	
*P*(T<=t) two-tail	0.04534*		0.00045 **	
t Critical two-tail	2.02107		2.01174	

^#^Allele A represent K099 allele and Allele B represent Fendou 16 allele. * and ** shows significant at P < 0.05 and P < 0.001 in Welch’s t-test, respectively.

### 3.7 *CEP-*like genes in soybean

**Figure 5:**
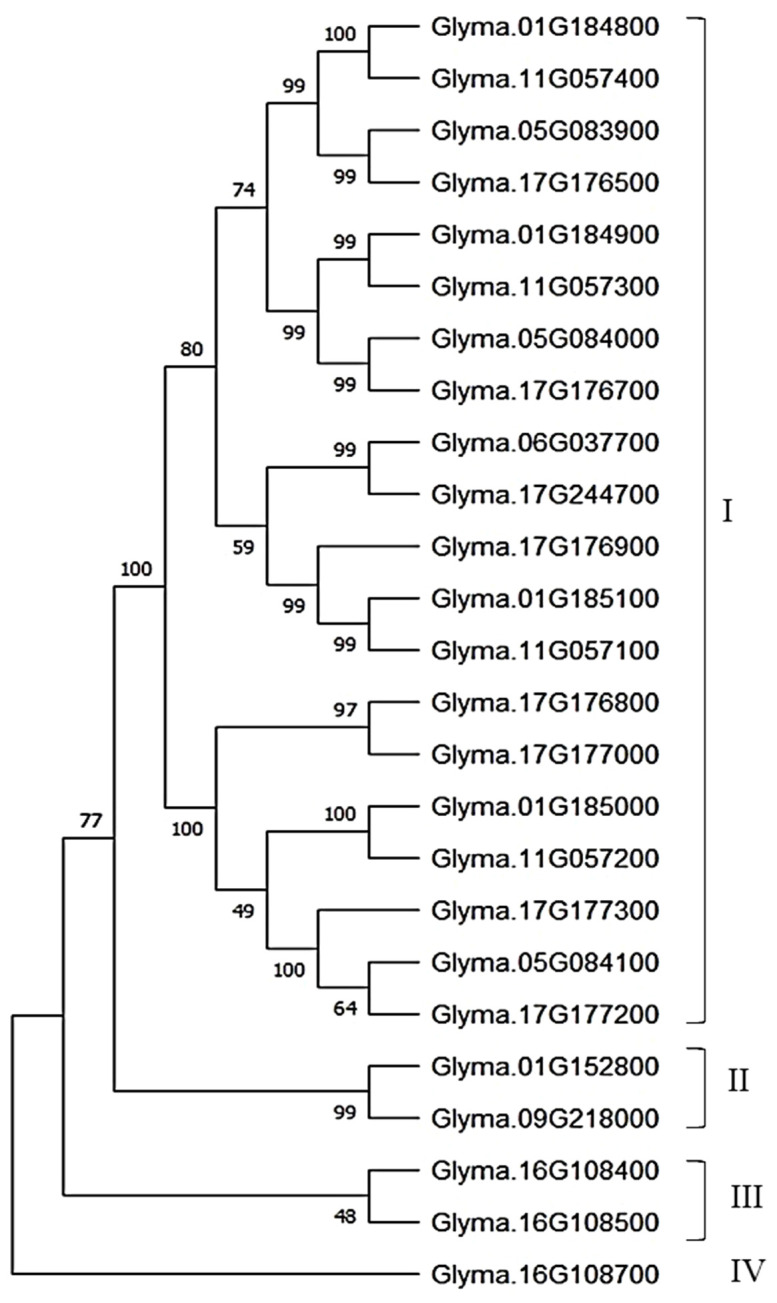
Phylogenetic tree of the protein sequence of 25 *CEP-*like genes in *Glycine max.*.

**Figure 6:**
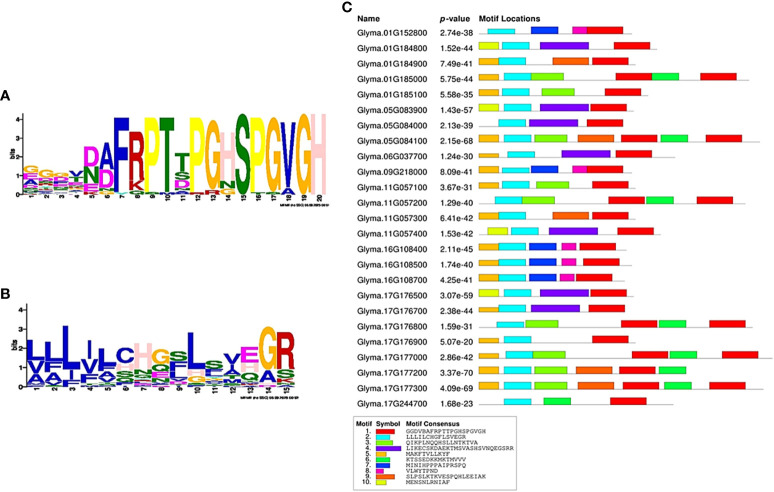
Consensus motif pattern among 25 CEP-like proteins in *Glycine max*. **(A)** Weblogo of the CEP consensus motif (Motif 1), **(B)** Weblogo of the N-terminal signal peptide consensus motif (Motif 2), **(C)** Consensus motif composition of 25 CEP-like proteins detected by MEME Suite.

A total of 25 *CEP*-like genes were identified that contain an N-terminal signal peptide and the CEP-like conserved motif ‘FRPToPGHSPGVGH’ (at consensus cutoff of 70%). These 25 *CEP-*like genes were distributed on seven chromosomes. Except for Chr.06 and 09, other *CEP-*like genes were located in tandem, suggesting that their copy number increased by tandem duplication. The highest tandem copies (eight) were present on Chr. 17. Phylogeny analysis using the neighbor-joining clustering method showed that most CEP-like proteins were clustered together in group I, while two CEP-like proteins from Chr.01 and Chr.09 were grouped in cluster II. The two CEP-like proteins of *Glyma.16g108400* and *Glyma.16g108500* from Chr.16 were clustered in group III, whereas the CEP protein from *Glyma.16g108700* was most divergent (**Figure 5**). Consensus motif analysis using MEME Suite identified consensus motif distribution patterns among 25 CEP proteins with an N-terminal signal peptide motif having only one site for each sequence, while a C-terminally encoded peptide conserved motif (Motif 1) was present as a single copy in 19 members and as two copies in six members (**Figure 6**). Two conserved domains (VLWYTPND and MINIHPPPAIPRSPQ) are specifically found in three *CEP-*like genes of Chr.16 and two members of group II of the phylogenetic tree, that is, *Glyma.01G152800* and *Glyma.09G218000* (**Figure 6**). Three proline residues were present in the 15 amino acid CEP domain of *Glyma.16g108500*, while only two proline residues were present in the CEP domain of *Glyma.16g108700*.

### 3.8 Synthetic peptide experiments

**Figure 7:**
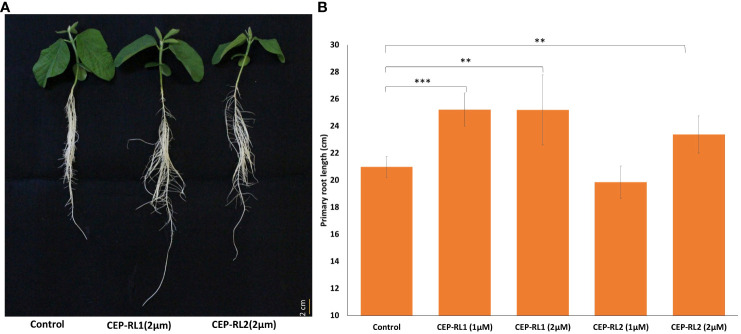
Effect of synthetic C-terminally encoded peptides (CEPs) on the development of the primary root length in K099, **(A)**. Shoot and root phenotypes of control and CEP treated plants (0. 5× Hogland solution pH 6.5), **(B)**. The difference in primary root length between control and CEP-treated plants (*n* = 8). *** and ** indicate significant differences at *P* < 0.001, and *P* < 0.01, respectively, as identified by the Student’s t-test.

Because the analysis of gene expression in contrasting germplasm excluded *Glyma.16g112400* and *Glyma.16g108400* from the possibility of their positive role in regulating the development of primary root length, we focused on *Glyma.16g108500* and *Glyma.16g108700* for further studies using 15 amino acid synthetic peptide of the predicted CEP domain for these genes. Two different concentrations of two CEPs for these genes (CEP-RL1 and CEP-RL2) were applied in Hoagland solution-based hydroponic culture (pH 6.5) for the short-rooting genotype K099. The results of this experiment showed that the length of the primary root was significantly higher for CEP-RL1 at concentrations of 1 µM and 2 µM s, whereas for CEP-RL2, the length of the primary root was significantly higher at a concentration of 2 µM concentration, compared to the control (**Figure 7**). Analysis of the effect of CEP treatment on total root length and root tips showed that at higher concentrations of each CEP (2 µM), total root length and root tips were significantly lower in CEP-RL1 treatment, but not in the case of CEP-RL2 treatment (**Supplementary Figure 2**).

### 3.9 Tissue-specific gene expression analysis

**Figure 8:**
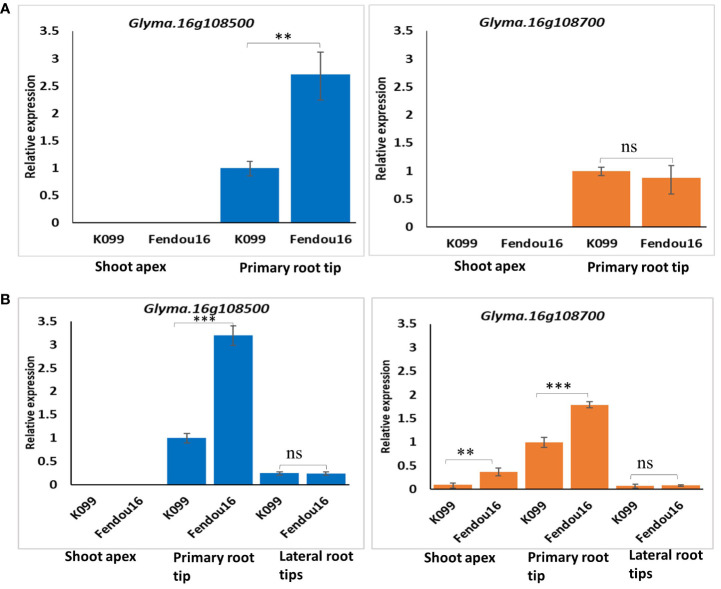
Relative expression of *Glyma.16g108500* and *Glyma.16g108700* in K099 and Fendou 16, **(A)** 4-day tissues, **(B)** 7-day tissues. *GmActin* was used as a normalizer. Significant differences between K099 and Fendou 16, are marked with asterisks (**P<0.01; ***P<001; ns, not significant; Student’s t-test).

In order to observe the expression pattern of two CEP genes in meristematic tissues of parental genotypes, the shoot apex and primary root tip were analyzed 4-day after sowing. Lateral roots appeared properly after 6–7 days of sowing, and so the shoot tip, the primary root tip, and the lateral root tips were analyzed at the 7-day stage. The analysis of gene expression for *Glyma.16g108500* in the shoot and the primary root tip of 4-day seedlings showed expression only in the primary root tip, where the expression was 2.7-fold higher in Fendou 16 than in K099 (**Figure 8A**). For *Glyma.16g108700*, expression was observed in the primary root, but the expression level was similar in both genotypes (**Figure 8A**). No expression was observed in shoot apex for either gene. These results indicated that the genes *Glyma.16g108500* and *Glyma.16g108700* are root-specific in the early stage of root development. In the primary root and lateral root tissues of 7-day seedlings, both genes showed expression and the expression level was higher in the primary root than in the lateral roots (**Figure 8B**). Fendou 16 showed a 3.2-fold higher expression of *Glyma.16g108500* and 1.8-fold higher expression of *Glyma.16g108700* in primary root than K099, but no differential expression was observed in lateral roots. In the shoot apex of 7-day seedlings, no expression was observed for *Glyma.16g108500*, but low expression was observed for *Glyma.16g108700*. These results also showed the involvement of these two genes in the early stage of primary root development.

## 4 Discussion

Genes controlling root traits are difficult to characterize due to the difficulty in phenotyping root traits under field conditions. Furthermore, roots exhibit high plasticity even under slight changes in the growing environment, making accurate phenotyping more difficult. Genetic architecture of root architecture traits in soybean has been dissected in some studies by root phenotyping under hydroponic, semi-hydroponic and field conditions, and quantitative trait loci (QTLs) mapping (Abdel-Haleem et al., 2011; Brensha et al., 2012; Liang et al., 2014; Manavalan et al., 2015; Prince et al., 2015; Yang et al., 2019; Prince et al., 2020; Chen et al., 2021; Wang et al., 2022). In our previous study a major locus for primary root length, *qRL16.1*, was mapped on Chr.16 explaining 30.25% of the total phenotypic variation under hydroponic culture (Chen et al., 2021). Traditionally, genes underlying QTLs were cloned and characterized using map-based cloning, which is a time-consuming process. However, in the next-generation sequencing (NGS) era and post NGS-era, gene identification and characterization are much faster (Jaganathan et al., 2020). We used an approach similar to bulk segregant RNA Seq (BSR-Seq) for gene discovery (Liu et al., 2012). In our current study, complete transcriptome sequencing of contrasting NILs along with their parents was performed to identify DEGs in the *qRL16.1* genomic region. The DEGs identified in this way were further analyzed for their association with the primary root length trait in contrasting germplasm using RT-PCR and marker-trait association analysis. The germplasm accessions of the high rooting group in the contrasting germplasm set had an almost two-fold difference in primary root length compared to the low rooting group. The contrasting NILs and advanced backcross lines pairs showed differential expression for all four genes tested; however, the contrasting germplasm accessions set showed differential expression only for two genes, *Glyma.16g108500* and *Glyma.16g108700;* this narrowed down further work to these two genes only. The SNP and Indel polymorphisms, RL1SNP968_T/C and RL1INDEL363_–/TA, present upstream of *Glyma.16g108500* had identical genotypes in all germplasm analyzed and constitute a haplotype. The frequency of the Fendou16 allele was higher in this haplotype and it showed a significant association with the length of the primary root and total roots. Finally, the function of these genes was validated by external treatment of synthetic peptides of the CEP domains of the two secretary peptide genes, which demonstrated their effect on primary root length.

Secreted regulatory peptides are signaling molecules that arise from genes that typically encode an N-terminal secretion signal and one or more conserved peptide domains (Delay et al., 2013). They are involved in many aspects of the development of shoots and roots. The conserved C-terminal domains of *Glyma.16g108500* and *Glyma.16g108700* are similar to the conserved domains of Group-I *CEP*-like genes in *Arabidopsis* (Roberts et al., 2013). The *CEP* gene family is known for its role in root length development and nutrient response in Arabidopsis (Ohyama et al., 2008; Delay et al., 2013; Roberts et al., 2013). The two *CEP*-like genes identified in this study for root development in soybean have a conserved C-terminal domain and an N-terminal signal peptide for the secretory pathway. Interestingly, in the case of *Arabidopsis CEP*-like genes, overexpression or external application of CEPs has shown reduced root growth (Ohyama et al., 2008; Roberts et al., 2013). In Arabidopsis, CEPs reduce the length of the primary root by slowing growth and reducing the lateral root density prior to the initiation of the lateral root (Delay et al., 2013).

Studies on the negative effect of increasing the concentration of CEP on root development have been reported in *Arabidopsis* (Ohyama et al., 2008; Delay et al., 2013; Roberts et al., 2013), Apple (Yu et al., 2019) and *Medicago* (Imin et al., 2013); however, our study is the first report on the positive effect of a higher concentration of CEPs on root length. During evolution, the soybean genome has undergone two rounds of genome duplication events, and subsequent gene duplication and neofunctionalization resulted in the acquisition of new functions for many genes (Schmutz et al., 2010). This may be the reason why higher expression of *CEP*-like genes is a positive regulator of root length in soybean. The number of *CEP* genes in soybean is also higher than that reported in any other species (Delay et al., 2013; Imin et al., 2013; Roberts et al., 2013; Yu et al., 2019; Aggarwal et al., 2020). There may be a distinct and important role for different CEPs in soybean. Further experiments on CEP overexpression, gene knockout using gene editing, and external application of different *CEP*-like proteins in soybean may reveal their functionality.

In conclusion, transcriptome analysis, gene expression studies in contrasting NILs and germplasm, marker-trait association in germplasm, and synthetic peptide assay provided strong evidence for the involvement of the two *CEP-*like genes *Glyma.16g108500* and *Glyma.16g108700* in the regulation of root length in soybean. These genes will be useful for soybean improvement toward developing a deep and robust root system to withstand low moisture and nutrient regimes.

## Data availability statement

The datasets presented in this study can be found in online repositories. The names of the repository/repositories and accession number(s) can be found in the article/**Supplementary Material**.

## Author contributions

GK: Writing – original draft, Investigation, Formal analysis. DC: Writing – review & editing, Investigation, Formal analysis. CP: Writing – review & editing, Investigation. DX: Writing – review & editing, Supervision, Funding acquisition, Conceptualization.
