# Supplementary material for: C-terminally encoded peptide-like genes are associated with the development of primary root at qRL16.1 in soybean
Source: Front Plant Sci. 2024 Apr 15;15:1387954. doi: 10.3389/fpls.2024.1387954 (PMC11056954; doi:10.3389/fpls.2024.1387954)
Supplement: Supplementary file 1 [file DataSheet_1.pdf]

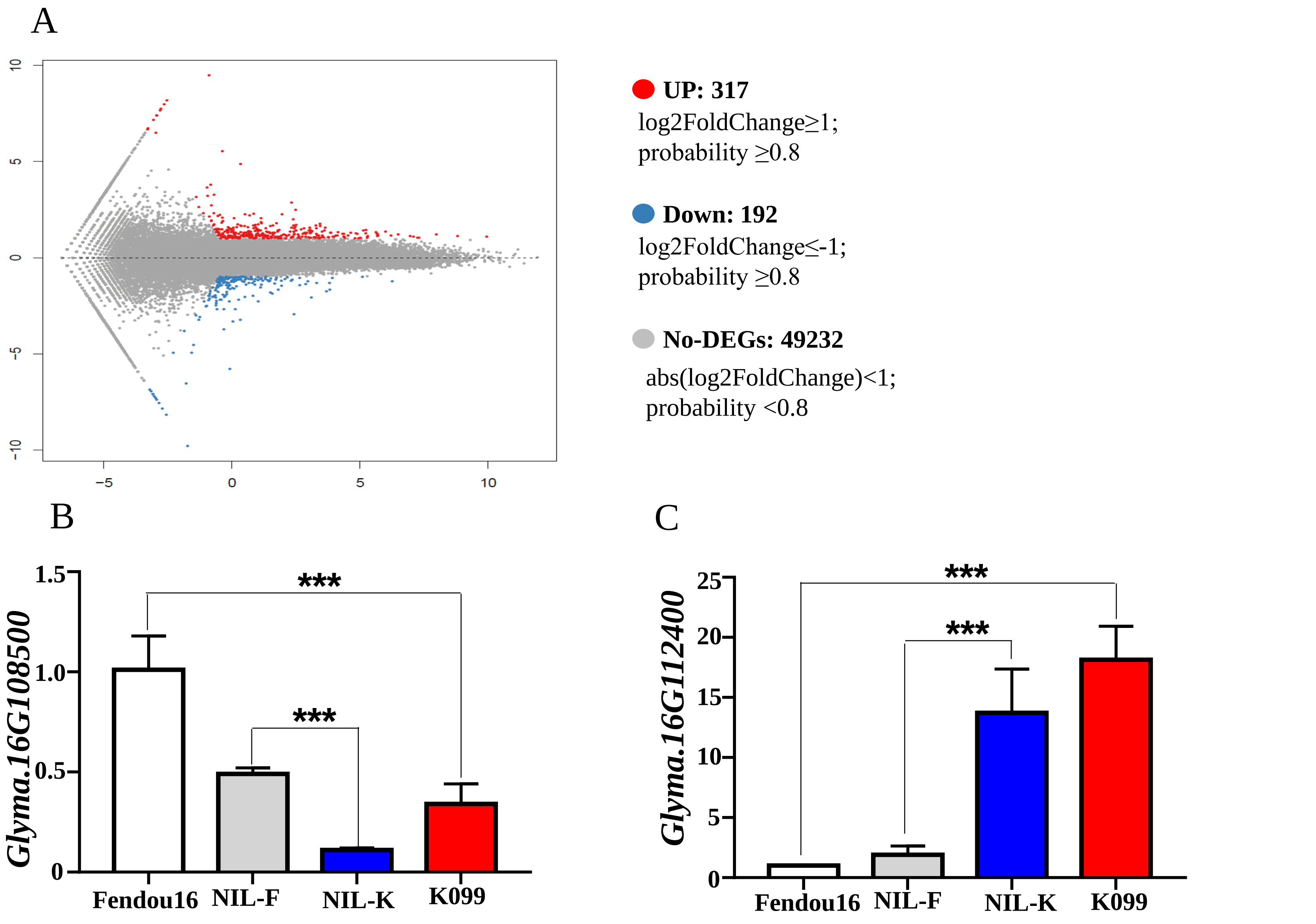

Supplementary Figure 1. RNA-Seq analysis for root transcriptome of NIL-F and NIL-K. A. Upregulated and downregulated genes, B. Relative expression *of* *Glyma.16G108500* in two parents (Fendou 16 and K099) and two NILs (NIL-F and NIL-K), C. Relative expression *of* *Glyma.16G112400* in two parents (Fendou 16 and K099) and two NILs (NIL-F and NIL-K). Statistically significant differences between K099 and Fendou16, NIL-K and NIL-F, are marked with asterisks （***$P < 0.001$; Student's t-test).

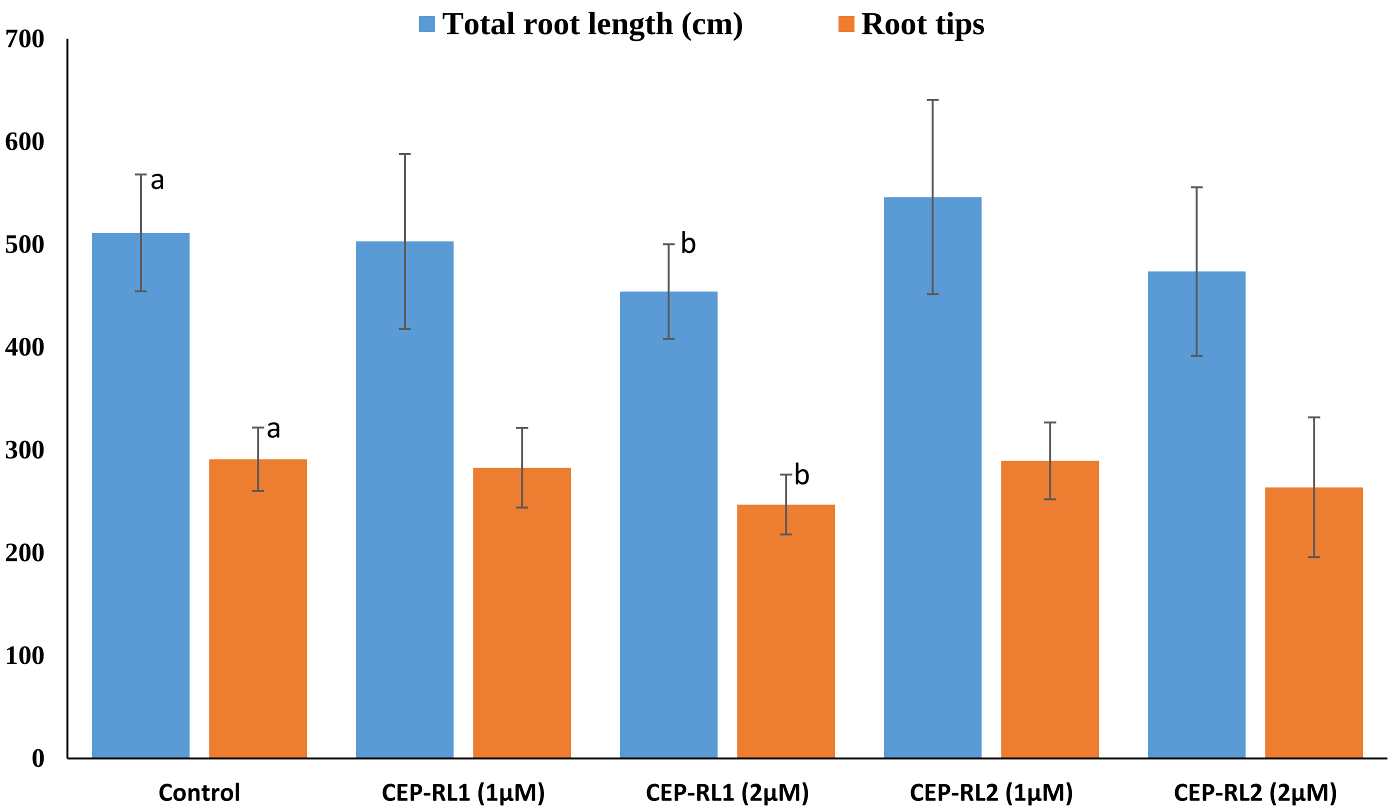

Supplementary Figure 2. Effect of synthetic C-terminally encoded peptides (CEPs) on total root length and root tips of K099, (0. 5× Hogland solution, pH 6.5), letters a and b indicates significant difference at $P < 0.05$, as identified by Students *t*-test.
